# Molecular Mechanisms of ARD1 in Tumors

**DOI:** 10.1002/cam4.70708

**Published:** 2025-03-03

**Authors:** Chunjiao Yu, Hongtao Lei, Xuefei Hou, Shan Yan

**Affiliations:** ^1^ Institute of Biomedical Engineering Kunming Medical University Chenggong District, Kunming Yunnan People's Republic of China; ^2^ Yunnan Key Laboratory of Breast Cancer Precision Medicine Chenggong District, Kunming Yunnan People's Republic of China

**Keywords:** acetylation, ARD1, tumor

## Abstract

**Introduction:**

Arrest‐deficient protein 1 (ARD1) is an acetyltransferase that acetylates the N‐terminal amino acids and internal lysine residues of proteins. It plays a crucial role in various cellular processes. The significance of ARD1 in tumor development has become increasingly evident in recent years.

**Methods:**

This review analyzes the regulatory role of ARD1 in tumor progression by examining its involvement in processes such as cell cycle regulation, cell proliferation, metastasis, apoptosis, and autophagy. Additionally, we discuss the expression patterns and molecular mechanisms of ARD1 in different types of cancer.

**Results:**

Elevated levels of ARD1 have been reported in several cancer types. Its increased expression is associated with various tumor characteristics, suggesting it may serve as a potential prognostic biomarker. Furthermore, ARD1 could be targeted for the development of novel cancer therapies.

**Conclusion:**

Understanding the role of ARD1 in tumor biology provides valuable insights into potential therapeutic targets and biomarkers for cancer treatment. This review highlights the advances in ARD1‐related research and suggests that it may be a promising avenue for improving cancer prognosis and treatment strategies.

## 1 Introduction

Acetylation is a primary post‐translational modification of eukaryotic proteins, playing a crucial role in various life activities [1, 2]. Dysfunctional acetylation contributes to diseases such as neurodegeneration, atherosclerosis, aneurysm, myocardial infarction, and tumors [3]. ARD1 is an acetyltransferase that catalyzes the post‐translational modification of proteins. Research has revealed that ARD1's normal physiological function is closely associated with the cell cycle, oxidative stress, autophagy, development, proliferation, and migration [4, 5, 6, 7]. Mutations in ARD1 lead to cell cycle arrest, impaired cell growth, and spore germination, indicating a pivotal role in controlling the mitotic cycle and cell survival [8]. Although the role of ARD1 in numerous tumors has been reported, its specific role and regulatory mechanism remain unclear. Considering the widespread acetylation modifications in organisms, there is ample research opportunity to explore both known and unknown functions of ARD1 [9, 10, 11]. Here, we review the progress of research on the biological functions and molecular mechanisms of ARD1 in tumors, which will provide new perspectives for an in‐depth understanding of the application of ARD1 in future clinical practice.

## 2 Acetylation Modification, Structure and Function of ARD1

### 2.1 Acetylation Modification

**FIGURE 1:**
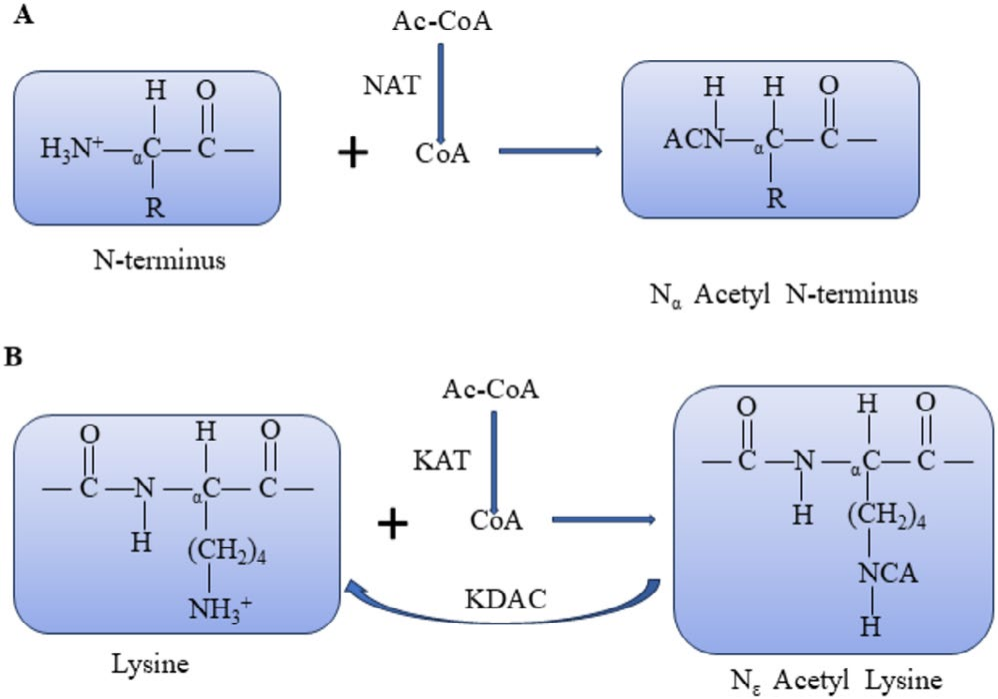
N‐terminal and internal lysine acetylation by ARD1. (A) ARD1 acetylates the α‐amino group of the N‐terminal residues of newly synthesized proteins. The acetyl group (red) is transferred from acetyl coenzyme A to the N‐terminal α‐amino group. (B) ARD1 also acetylates the ε‐amino group of lysine residues within proteins, and lysine deacetylases can reverse lysine acetylation. NAT, N‐terminal acetyltransferase; KAT, lysine acetyltransferase; KDAC, lysine deacetylase.

Acetylation comprises Nα‐terminal acetylation (Nα‐Ac) and Nε‐terminal acetylation (Nε‐Ac). Nα‐Ac involves the covalent binding of acetyl groups from acetyl coenzyme A to the α‐residue at the N‐terminal end of the nascent peptide chain, catalyzed by N‐terminal acetyltransferase enzymes (NATs) (Figure 1A). Conversely, Nε‐Ac entails the covalent binding of acetyl groups from acetyl coenzyme A to the ε‐amino group of the lysine residue, catalyzed by lysine acetyltransferases (HATs/KATs) (Figure 1B) [12]. Nα‐Ac is prevalent in eukaryotes, particularly humans, with an incidence exceeding 80%–90%. Thisacetylation primarily takes place during the cotranslation of new peptide chains exceeding 20 amino acids emerging from the ribosome. Additionally, it occurs in post‐translational modification, which is irreversible [13, 14]. In contrast, Nε‐Ac predominantly occurs during post‐translational modification, and this process is reversible. Lysine residues undergo modification through acetylation facilitated by KATs or by removing the acetyl group from the lysine residue, catalyzed by lysine deacetylases (KDACs) [15]. This dynamic equilibrium significantly influences the regulation of gene transcription, cellular signaling, metabolism, apoptosis, and development [16]. Notably, Nε‐Ac neutralizes the formation of positive amino acids in the amino group. Nε‐Ac counteracts the positive charge formed on the amino group, impacting biological processes such as protein conformation, protein‐DNA interactions, and protein–protein interactions [15].

### 2.2 ARD1 Structure

In 1985, Whiteway et al. first identified ARD1 in *Saccharomyces cerevisiae* , while Mullen et al. confirmed its NAT activity in 1989. Subsequently, they uncovered that ARD1 exhibits both NAT and KAT activities in mammals [17]. ARD1 catalyzes the acetylation of the α‐residue at the N‐terminal end of the nascent peptide chain and the Nε‐terminal end of the lysine residue, including self‐acetylation [18, 19].

**FIGURE 2:**
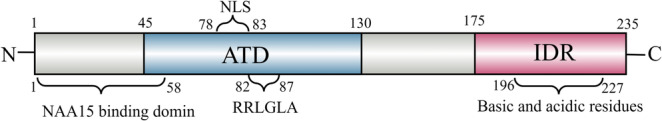
Domain structure of ARD1. Human ARD1 protein is composed of 235 amino acids. The N‐terminal acetyltransferase domain (ATD) is located between amino acids 45–130, and NLS interacts with nuclear carriers to transport ARD1 into the nucleus. The N‐terminal region of ARD1 is a key region that binds to NAA15, and the C‐terminal region of amino acids 175–235 is predicted to be an intrinsically disordered region (IDR).

ARD1, also known as NAA10, weighs 26,459 Da and is present in the cytoplasm and nucleus [17]. The murine *ARD1* gene on chromosome XA7.3 has nine exons and two variants: mNaa10235 and mNaa10225. In contrast, the human *ARD1* gene on chromosome Xq28 has eight exons and three variants: hNaa10235, hNaa10225, and hNaa10229. The different variants, specifically hNaa10235, hNaa10220, and hNaa10229, exhibit varying amino acid counts but share high conservation at the N‐terminus. Human Naa10235 consists of 235 amino acid residues, and its N‐terminus spans amino acids 1 to 178, forming a stable 3D globular domain [20] crucial for ARD1's function. Amino acids 1 to 58 are binding sites for the homologous protein NAA15. The acetyltransferase activity concentrates in the region spanning amino acids 45 to 130(Figure 2). Notably, amino acid residues 82 to 87 (RRLGLA) function as core sites for the enzyme's collaboration with the acetyl cofactor. Mutation of the 82nd amino acid residue from R to A significantly reduces the acetyltransferase activity, and mutation of the 85th amino acid from G to A partially deactivates it [21]. The C‐terminus of ARD1, encompassing amino acids 179–235, exhibits α‐helix and β‐folding structures, indicating its flexible non‐structural nature [22]. This region serves as a site for interaction with other proteins, forming complexes or undergoing post‐translational modifications for specific functions [23].

### 2.3 ARD1 Function

**FIGURE 3:**
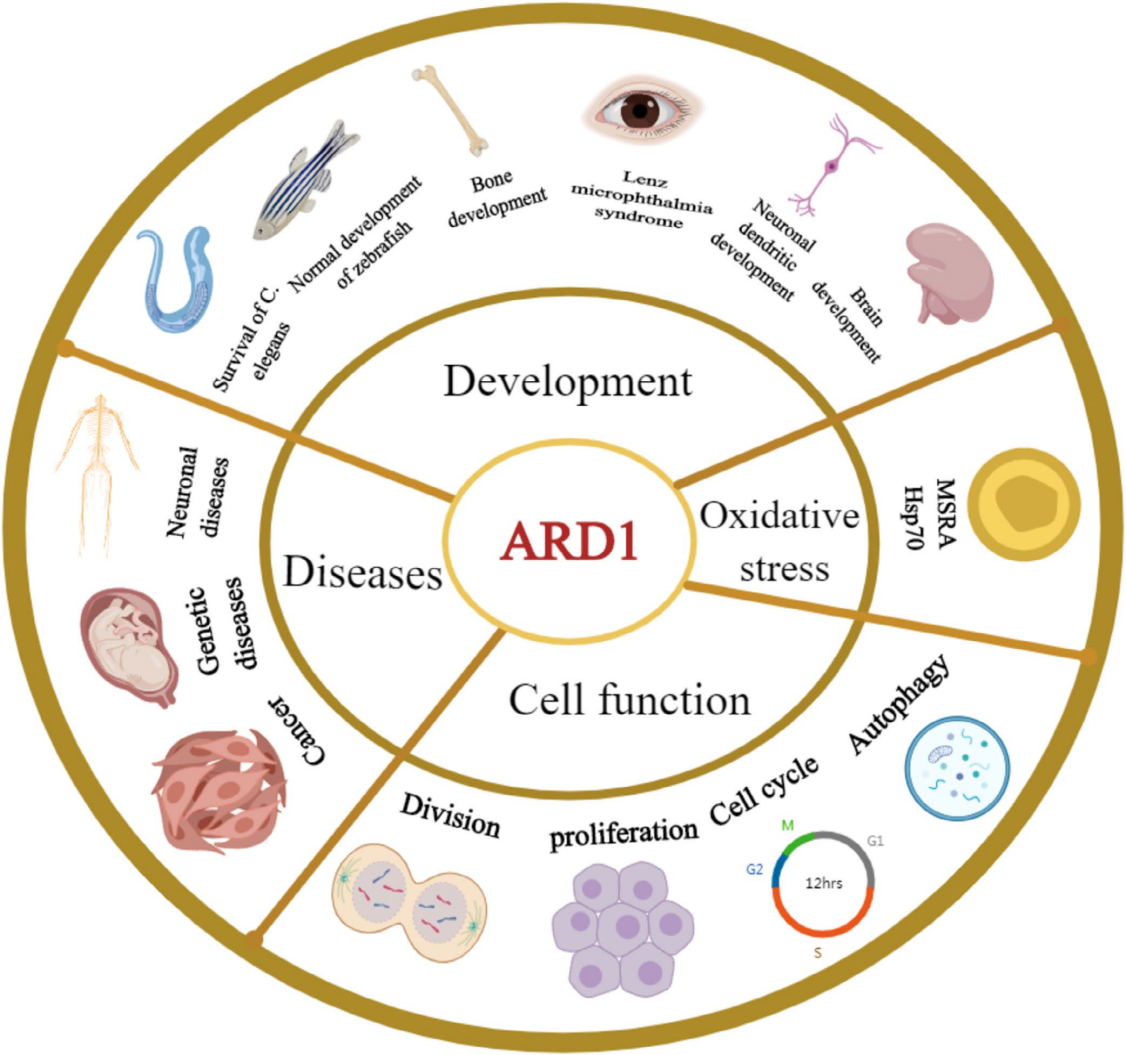
Shows the biological functions of ARD1. ARD1 is involved in various functions, including development, cellular function, oxidative stress, and a number of related diseases.

ARD1 is integral to various cellular functions, governing cell division, proliferation, and tumorigenesis. Additionally, it plays a crucial role in normal development and survival, including brain development, particularly in the formation of neuronal dendrites, potentially influencing neurological diseases. ARD1 is also implicated in susceptibility or protection against oxidative stress (Figure 3).

ARD1 is a critical enzyme extensively involved in regulating the cell cycle, metabolism, and cellular homeostasis. ARD1 profoundly affects cell proliferation, apoptosis, and oxidative stress responses by acetylating various substrates. For instance, ARD1 regulates dNTP levels by acetylating the K405 residue of SAMHD1, thereby promoting the G1/S transition and supporting cell growth [24]. Additionally, its dual regulatory role in oxidative stress is evident in its acetylation of heat shock protein 70 (Hsp‐70) and methionine sulfoxide reductase A (MSRA), contributing to cellular homeostasis and stress sensitivity, respectively [25].

ARD1 also plays a pivotal role in autophagy and metabolic regulation. Under hypoxic and amino acid‐deprived conditions, ARD1 facilitates the formation of autophagosomes by regulating Beclin1 phosphorylation and promoting PtdIns3P synthesis, thereby maintaining cellular homeostasis [26]. Furthermore, ARD1 demonstrates dynamic changes during embryonic development, with high expression in specific tissues critical for organogenesis [27, 28]. For example, ARD1 expression peaks during the E12 –E14 embryonic stages in murine kidneys, livers, and lungs and decreases significantly postnatally [29]. Notably, ARD1 acetylates the K225 residue of Runx2, disrupting its interaction with CBFβ, thereby influencing bone development. ARD1 knockout mice exhibit severe developmental defects, including growth retardation and fetal malformations [30].

In summary, ARD1 plays a central regulatory role in various cellular and biological processes, providing a significant foundation for further exploration of its functions in cancer.

## 3 ARD1 Regulation at Various Molecular Levels

Both transcription factors and epigenetic modifications regulate the expression of ARD1. Recent studies have shown that the androgen receptor (AR), upon binding to androgens, recruits RNA polymerase II by binding to specific DNA sequences, thereby promoting the transcription of ARD1. This mechanism establishes a positive feedback loop that plays a crucial role in prostate tumorigenesis [31]. Furthermore, epigenetic modifications, such as the methylation status of the ARD1 promoter, also regulate ARD1 expression. Notably, hypomethylation observed in lung cancer may enhance ARD1 expression, contributing to tumor progression [32].

Post‐transcriptional regulation predominantly involves RNA‐binding proteins and microRNAs. For instance, miR‐342‐5p and miR‐608 inhibit tumorigenesis in colon cancer cells by targeting ARD1 mRNA degradation. This indicates that ARD1 expression can be regulated post‐transcriptionally via miRNA‐mediated mRNA degradation [33]. Furthermore, Gao et al. discovered that RGMB‐AS1 binds to the amino acid 82–87 region of ARD1, activates its acetyltransferase activity, and promotes the conversion of acetyl‐coenzyme A to HMG‐CoA, thus influencing ferroptosis. These findings suggest that post‐transcriptional regulation of ARD1 extends beyond degradation and may also involve functional activation [34].

Post‐translational modifications of ARD1, such as self‐acetylation, directly influence its enzymatic activity and interactions with other cellular proteins. Zeng et al. identified residue K136 as a critical self‐acetylation site of ARD1, and the K136R mutation significantly impaired ARD1's ability to promote cancer cell proliferation. Self‐acetylation enhances cancer cell growth by activating the transcription factors β‐catenin and AP‐1, which induce downstream expression of cyclin D1 [35]. HYPK binds to ARD1, modulates its catalytic site, and inhibits its acetylation activity, suggesting that HYPK functions as a negative regulator capable of post‐translationally modulating ARD1's function. These post‐translational modifications render ARD1's role in cancer‐related processes, such as cell cycle progression, migration, and apoptosis, more complex and refined [36].

**FIGURE 4:**
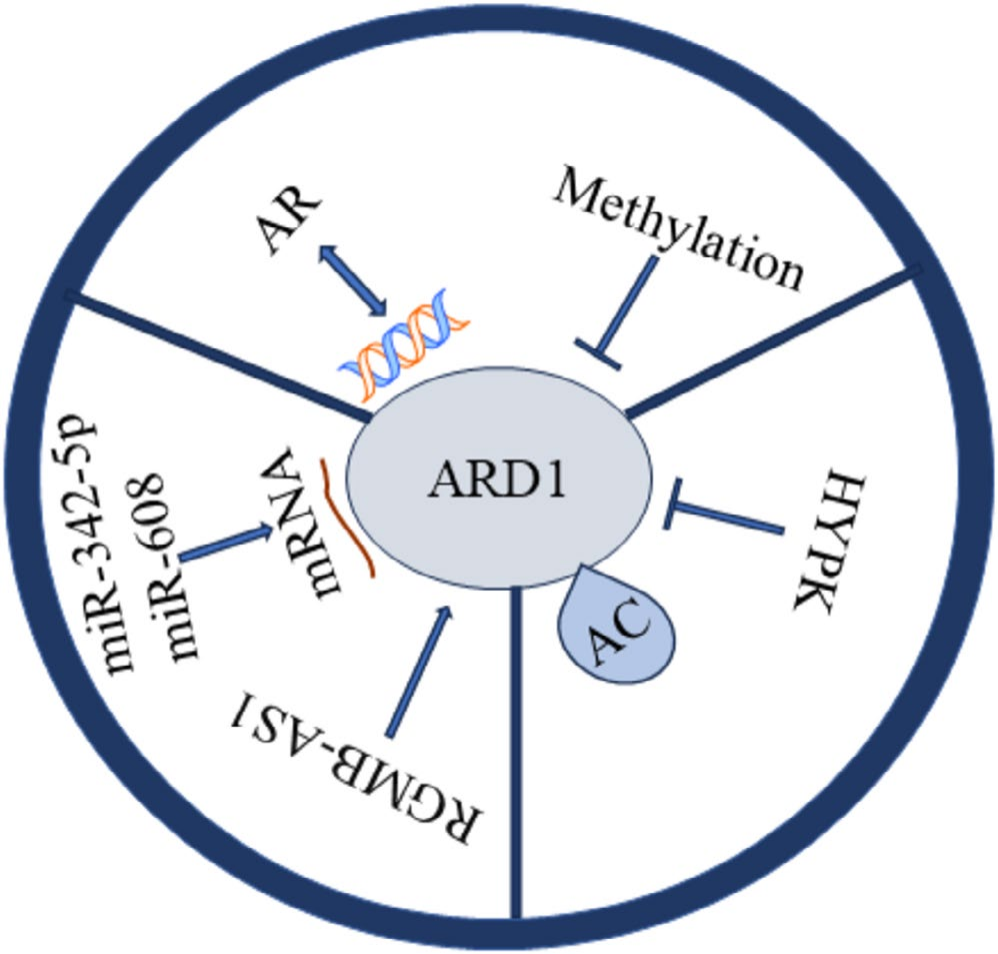
The molecular regulation of ARD1 at transcriptional, post‐transcriptional, and post‐translational levels.

By comprehensively understanding these transcriptional, post‐transcriptional, and post‐translational regulatory mechanisms, we can gain a better insight into the role of ARD1 in various types of cancers and provide theoretical support for the development of future ARD1‐based therapies (Figure 4). For instance, ARD1‐specific inhibitors or therapeutic strategies that interfere with AR –ARD1 interactions may be critical for the treatment of prostate cancer (PCa). At the same time, hypomethylation of ARD1 could serve as a potential prognostic biomarker for lung cancer.

## 4 ARD1 And Cancers

The role of ARD1 has been reported in many tumors, including breast, liver, osteosarcoma, lung, prostate, esophageal, renal, neurogliomas, oral squamous, and colorectal cancers. The role of ARD1 in tumors is controversial, as it can play both pro‐cancer and anti‐cancer roles.

### 4.1 Molecular Mechanisms in Breast Cancer

**FIGURE 5:**
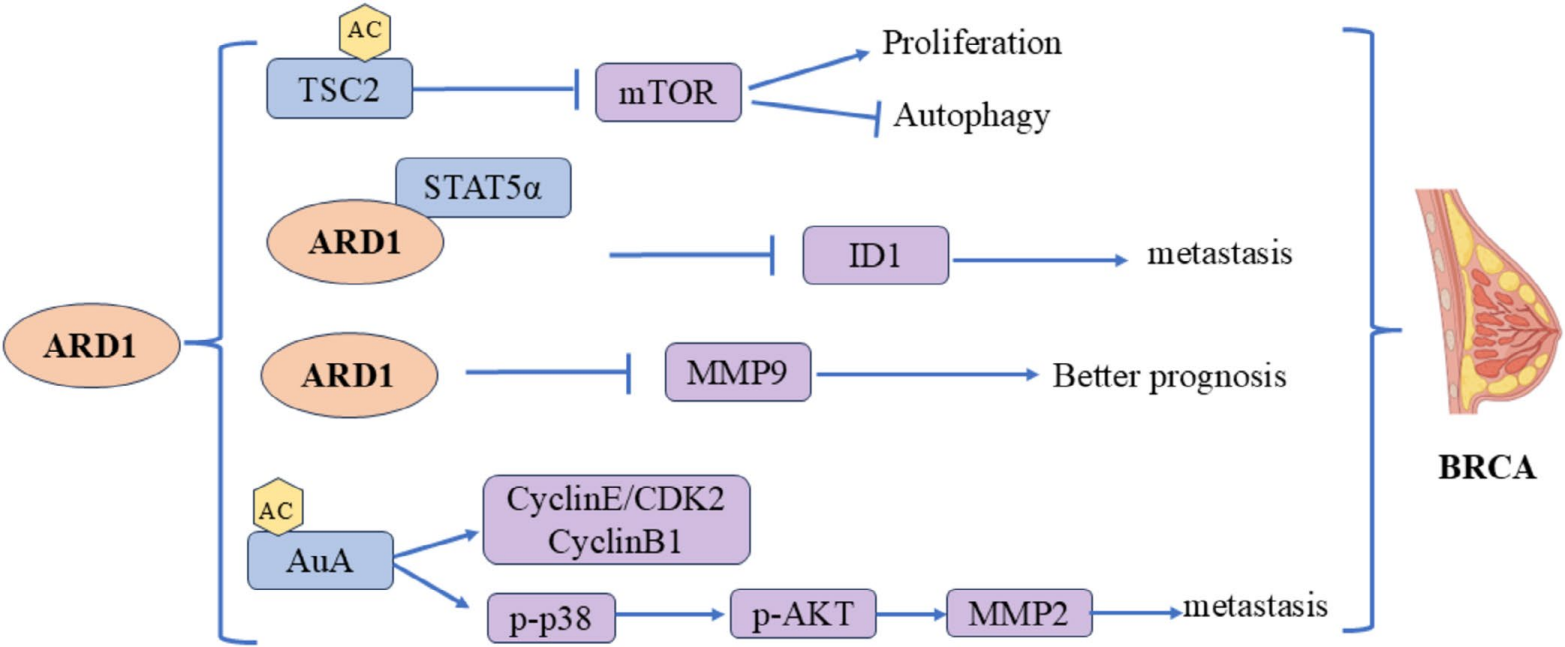
Molecular mechanisms of ARD1 in breast cancer.

Breast cancer (BRCA) is the most prevalent cancer among women. The role of ARD1 in breast cancer is complex and multifaceted, involving the regulation of various aspects such as cell proliferation, migration, and prognosis prediction (Figure 5). Zeng found that high ARD1 expression correlated positively with the survival rate of breast cancer patients and negatively with lymph node metastasis, indicating that ARD1 may enhance prognosis by inhibiting cancer cell migration and invasion. Specifically, ARD1 reduced breast cancer cells' migration and invasion capabilities in vitro. It slowed the growth and metastasis of xenografts in nude mice. Microarray screening further demonstrated that ARD1 downregulated the differentiation inhibitor ID1 and inhibited STAT5a‐stimulated ID1 expression by binding to the transcription factor STAT5α. Additionally, ARD1 antagonized the Janus kinase 2‐STAT5α signaling pathway by reducing p65‐activated IL‐1β expression, thereby inhibiting breast cancer cell metastasis [34]. Regarding prognosis, the relationship between ARD1 and MMP‐9 expression is notably significant. Regarding prognosis, the relationship between ARD1 and MMP‐9 expression is notably significant. High ARD1 expression was associated with better overall survival (OS) and disease‐free survival (DFS), whereas high MMP‐9 expression correlated with poorer prognosis. Combined analyses indicated that ARD1‐positive/MMP‐9‐negative patients exhibited the best OS and DFS, and multifactorial analysis confirmed the significant impact of ARD1 and MMP‐9 expression on breast cancer prognosis [37]. Wang et al. supported these findings, showing that upregulation of ARD1 was linked to a metastatic phenotype and poor prognosis in breast cancer [38]. Yu et al. examined the relationship between ARD1 and breast cancer by assessing its effect on cell proliferation. Their results suggested that ARD1 promotes the proliferation of breast cells [39]. Research shows that ARD1 can be acetylated at lysine residues 75 and 125 of AuA, significantly influencing AuA's kinase activity. When both K75 and K125 were mutated, AuA lost its kinase activity, markedly reducing its capacity to promote cell proliferation and migration. This implies that the acetylation status of AuA is essential for its biological functions. In terms of cell proliferation, acetylated AuA facilitates cell entry into mitosis and boosts the proliferation of breast cancer cells by activating cyclin E/CDK2 and cyclin B1. Furthermore, the acetylation of AuA enhances cell migration by activating the p38/AKT/MMP‐2 signaling pathway, facilitating cancer cell spread and metastasis. Thus, ARD1‐mediated AuA acetylation in breast cancer may serve as a crucial mechanism promoting tumor cell proliferation and migration [40]. mTOR is a critical cell growth and metabolism regulator closely linked to tumorigenesis and progression. Kuo et al. discovered that ARD1 inhibits mTOR activity by acetylating and stabilizing TSC2 through interaction with it, a regulatory mechanism that decreases mTOR activity. This regulatory mechanism diminishes cell proliferation and enhances autophagy, inhibiting breast cancer tumorigenicity. Furthermore, the abundance of ARD1 and TSC2 exhibited a significant correlation across various tumor types, particularly in breast cancer, where ARD1 levels notably influenced TSC2 stability and inhibition of the mTOR signaling pathway [41].

Different breast cancer cell lines are used in the study of Yu et al. and Kuo et al. [39, 41]. Breast cancer is a highly heterogeneous disease. Different cell lines represent different breast cancer subtypes with different genetic backgrounds and phenotypic characteristics. Therefore, the role of ARD1 in different cell lines may be different, resulting in different effects on cell proliferation and tumors. The population of breast cancer patients included in the study may have differences in disease stage, treatment history, genetic background, etc. These factors may affect the association between ARD1 expression and disease prognosis. ARD1 can both promote and inhibit the proliferation of breast cancer cells. This dual effect may also depend on the activation status of specific cell pathways and the existence of other regulatory factors. ARD1 activates or inhibits different pathways through the acetylation of specific target proteins, thus playing different roles in different biological backgrounds.

### 4.2 Molecular Mechanisms in Colorectal Cancer

**FIGURE 6:**
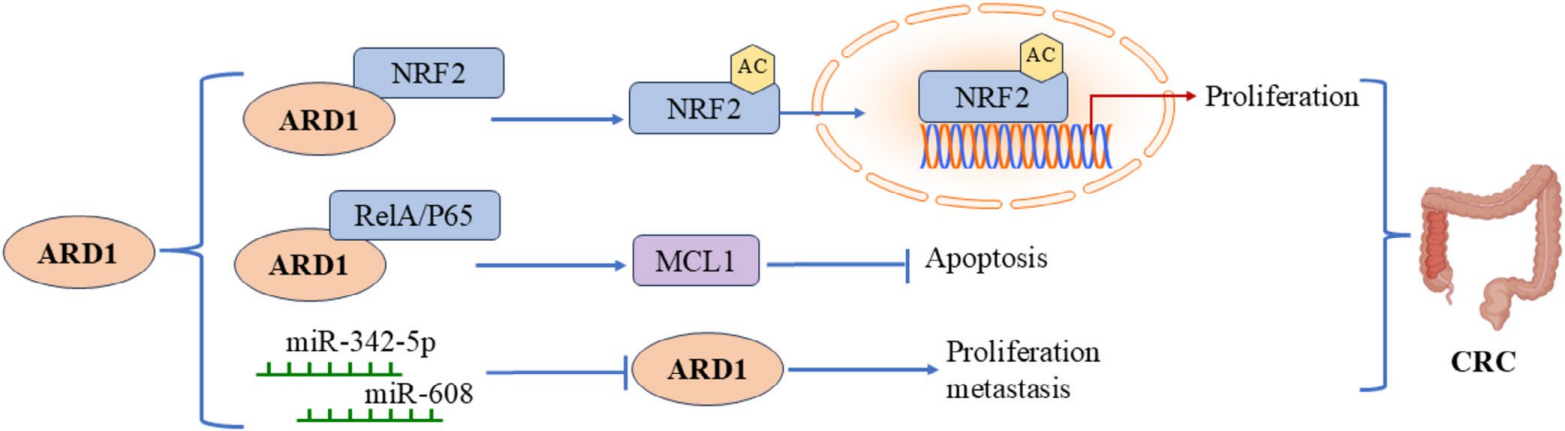
Molecular mechanisms of ARD1 in colorectal cancer.

Colorectal cancer (CRC) ranks as the third most common cancer worldwide. Previous studies have shown a connection between ARD1 and colorectal cancer, affecting various cellular processes, including proliferation, the cell cycle, and apoptosis (Figure 6). Yang et al. collected 33 pairs of human colon cancer samples alongside corresponding normal tissues. They found a significant increase in ARD1 expression in tumor tissues compared to adjacent normal tissues. Silencing ARD1 resulted in reduced tumorigenicity in colon cancer cells in vitro. miR‐342‐5p and miR‐608 were shown to regulate colon carcinogenesis by targeting ARD1 mRNA. Overexpression of miR‐342‐5p and miR‐608 significantly decreased both mRNA and protein levels of ARD1, inhibiting proliferation, migration, and cell cycle progression while promoting apoptosis in colon cancer cells. In an in vivo mouse xenograft model, these miRNAs significantly decreased the tumorigenicity of colon cancer cells, suggesting that miR‐342‐5p and miR‐608 inhibit proliferation and metastasis by downregulating ARD1 expression, highlighting the important role of miRNAs in regulating ARD1 and its downstream signaling pathways [33]. Jiang et al. reported that elevated ARD1 levels in colorectal cancer were associated with poor prognosis in colon cancer patients [42].

Similarly, Fang et al. performed immunofluorescence staining on 35 pairs of colon cancer and adjacent paracancerous tissues using an anti‐ARD1 antibody. They observed a notable upregulation of ARD1 expression in colorectal cancer tissues compared to normal colon tissues. Knockdown of ARD1 significantly decreased NRF2 protein levels without affecting its mRNA expression in human colon cancer cell lines, indicating that ARD1 may regulate NRF2 protein stability. Specifically, ARD1 overexpression enhanced NRF2 acetylation, and subsequent experiments and mass spectrometry analyses confirmed that ARD1 directly acetylates NRF2. Acetylated NRF2 was less prone to proteasomal degradation, preserving its stability and promoting its translocation and activation in the nucleus, enhancing its transcriptional activity and promoting colon cancer cell proliferation. This mechanism implies that ARD1 inhibits proteasome‐mediated NRF2 degradation by directly binding to and acetylating NRF2, thereby driving tumorigenesis and progression [43]. ARD1 also plays a role in colorectal cancer progression by regulating the expression of the anti‐apoptotic factor MCL1. Xu et al. discovered that ARD1 positively regulates MCL1 expression, and its deletion inhibits MCL1 expression, making cancer cells more sensitive to apoptotic stimuli. The underlying mechanism involves the physical interaction between ARD1 and RelA/p65. The ARD1‐RelA/p65 complex recruits to the p65 binding site in the MCL1 promoter region, activating MCL1 gene transcription. The positive correlation between MCL1 and ARD1 mRNA levels in colon and lung cancer tissues further supports the notion that ARD1 inhibits apoptosis by regulating MCL1 transcription. These findings underscore the potential of ARD1 as a biomarker for evaluating the prognosis of colorectal cancer [44].

### 4.3 Molecular Mechanisms in Lung Cancer

**FIGURE 7:**
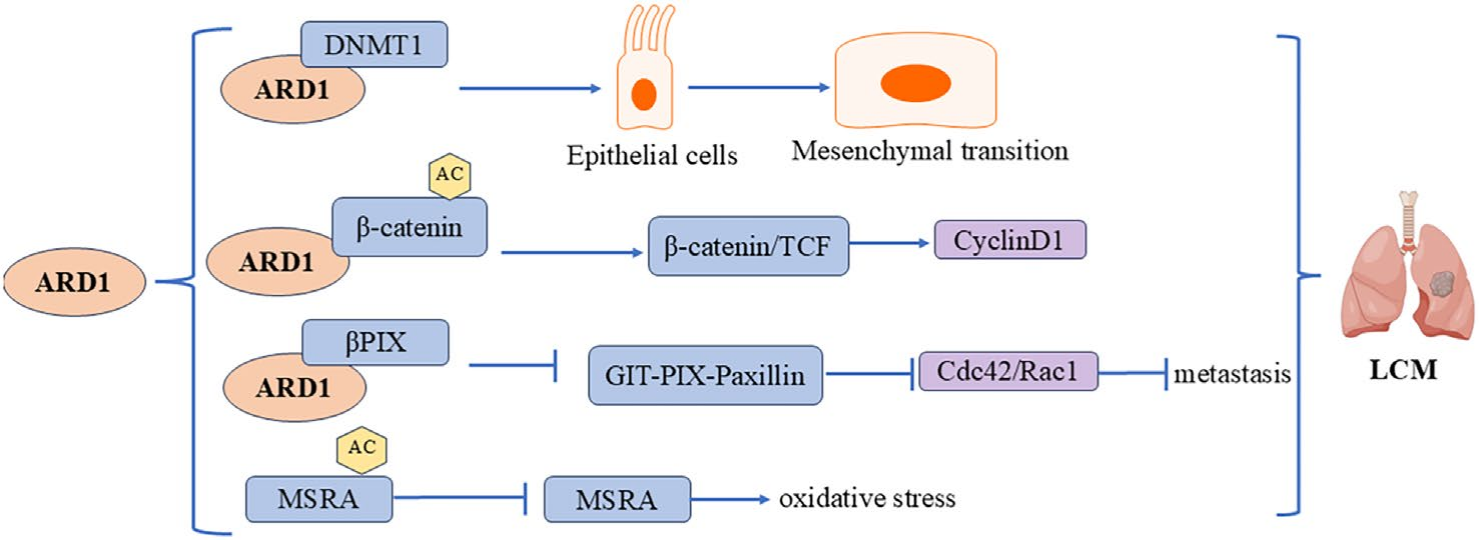
Molecular mechanisms of ARD1 in lung cancer.

Lung cancer is the leading cause of cancer‐related deaths in both men and women. The role of ARD1 in lung cancer is complex and multifaceted, involving mechanisms such as cell proliferation, transcriptional and epigenetic regulation, and responses to oxidative stress (Figure 7). Lee et al. performed a TCGA analysis, revealing significant upregulation of ARD1 in lung cancer tumor tissues compared to normal lung tissues. Their groundbreaking study identified aberrant methylation of the ARD1 promoter in non‐small cell lung cancer (NSCLC), suggesting ARD1 as a potential prognostic biomarker [32]. In lung cancer cells, ARD1 expression significantly impacts cell proliferation and cycle regulation. Lim's study found that silencing ARD1 inhibited the proliferation of H1299 and A549 lung cancer cells, leading to G1 phase arrest. This effect was partly mediated by downregulating CyclinD1 expression, a key regulator of the G1 phase, leading to cell cycle arrest. Further studies indicated that ARD1 knockdown reduced the transcriptional activity of CyclinD1 and inhibited the binding of the β‐catenin/TCF4 transcription factor to the CyclinD1 promoter. Specifically, ARD1 enhances the transcriptional activity of β‐catenin by promoting its acetylation, while ARD1 knockdown reduces β‐catenin acetylation, impacting CyclinD1 transcription. This suggests that ARD1 regulates CyclinD1 expression through β‐catenin acetylation, thereby influencing lung cancer cell proliferation [45]. Another study revealed that ARD1 is involved in lung carcinogenesis through its interaction with DNMT1, and overexpression of ARD1 correlates with poor survival in lung cancer patients. ARD1 promotes DNMT1 binding to DNA, particularly to tumor suppressor gene promoters (e.g., E‐cadherin), enhancing DNMT1's enzymatic activity and leading to decreased CpG methylation in the cadherin promoter region, thereby silencing the E‐cadherin gene. Silencing E‐cadherin contributes to lung cancer progression, highlighting ARD1's important role in regulating epigenetic modifications and tumorigenesis [46]. ARD1 also plays a significant role in regulating oxidative stress and cellular responses to oxidative damage. Research shows that ARD1 interacts with MSRA, specifically acetylating its K49 residue, inhibiting its enzymatic function. Under oxidative stress, ARD1 overexpression increased ROS levels, carbonylated proteins, and cell DNA breaks, promoting oxidative stress‐induced cell death. Studies with ARD1 transgenic mice revealed significantly higher oxidative tissue damage than wild‐type mice, further supporting ARD1's critical role in oxidative damage. These findings suggest a potential role for ARD1 as a regulator of MSRA in lung cancer, particularly in enhancing oxidative stress and promoting cell death [47]. Notably, Hua et al. reported contrasting findings in NSCLC. They observed lower ARD1 expression levels in metastatic lymph nodes compared to primary tumors. Patients with higher ARD1 expression exhibited more favorable prognoses and extended survival rates. ARD1 disrupts the assembly of the GIT‐PIX‐Paxillin complex by binding to the GIT‐binding region of PIX proteins, inhibiting Cdc42 and Rac1 activity, which significantly decreases cell migration and inhibits tumor growth and metastasis [48].

Lee et al. and Hua et al. The number of sample sources used in the study and the difference in the selection and sum of different samples may lead to inconsistent research results. NSCLC is a complex disease with multiple subtypes and significant genetic and phenotypic heterogeneity. ARD1 may play different roles in different subtypes or genetic backgrounds. The abnormal methylation of the ARD1 promoter region is mentioned in Lee et al. This suggests that the expression and function of ARD1 may be regulated by epigenetic modifications, which may vary in different patients or tumor samples, thus affecting the expression level of ARD1 and its correlation with prognosis. In addition, the prognosis of tumors is influenced by many factors, including, but not limited to, the molecular characteristics of tumors, the physiological conditions of patients, treatment regimens, etc. ARD1 expression may interact with other unrecognized biomarkers or clinical parameters. These complex interactions may lead to different results observed in different studies.

### 4.4 Molecular Mechanisms in Oral Squamous Cell Carcinoma

**FIGURE 8:**
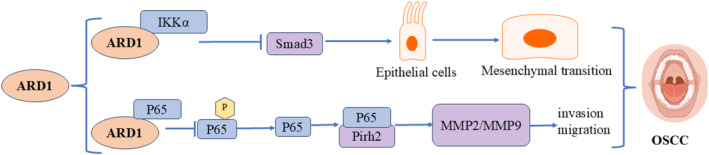
Molecular mechanisms of ARD1 in oral squamous cell carcinoma.

Oral squamous cell carcinoma (OSCC) is the most common type of oral cavity malignancy, classified as a subtype of head and neck squamous cell carcinoma (HNSCC) [49]. ARD1 exerts inhibitory effects in OSCC through multiple signaling pathways (Figure 8). Our previous study revealed elevated ARD1 expression levels in oral squamous carcinoma tissues. Its expression was inversely correlated with TNM stage, lymph node status, differentiation degree, and recurrence. Additionally, it inhibited the migration and invasion of oral squamous carcinoma cells [50]. Zheng et al. also observed ARD1 overexpression in oral squamous carcinoma tissues. They concluded that ARD1 positivity independently predicted a favorable prognosis for patients with oral squamous carcinoma [51]. Aberrant ARD1 expression is closely linked to OSCC progression, with its molecular mechanisms involving multiple signaling pathways, primarily the TGF‐β1/Smad and p53 pathways. In OSCC, ARD1 and IKKα show abnormal levels, and ARD1 influences epithelial‐mesenchymal transition (EMT) by regulating the TGF‐β1/Smad pathway. ARD1 was found to directly interact with IKKα and inhibit IKKα‐mediated activation of Smad3. Specifically, ARD1 blocked Smad3 transcriptional activation by IKKα upon TGF‐β1 stimulation, thereby reducing the expression of EMT‐related molecules. This mechanism restricts the migration, invasion, and EMT processes of OSCC cells by downregulating IKKα activity and Smad3 function. Thus, ARD1 inhibits OSCC progression, offering a novel intervention pathway to halt cancer cell invasion and metastasis [52]. Besides the TGF‐β1/Smad pathway, ARD1 inhibits OSCC invasion and metastasis by regulating the Pirh2‐p53 signaling pathway. Research demonstrated a physical interaction between ARD1 and RelA/p65, which blocked RelA/p65‐mediated transcriptional activation of Pirh2. Since Pirh2 is an E3 ubiquitin ligase that promotes p53 degradation, Naa10p's action elevated p53 levels by inhibiting Pirh2 activity. Elevated p53 levels further inhibited the expression of downstream targets such as MMP‐2 and MMP‐9, thereby reducing the invasive and metastatic capacities of OSCC cells. These findings suggest that ARD1 may play a crucial role in managing and treating OSCC, serving as a potential therapeutic target and prognostic marker [53].

### 4.5 Molecular Mechanisms in Prostate Cancer

**FIGURE 9:**
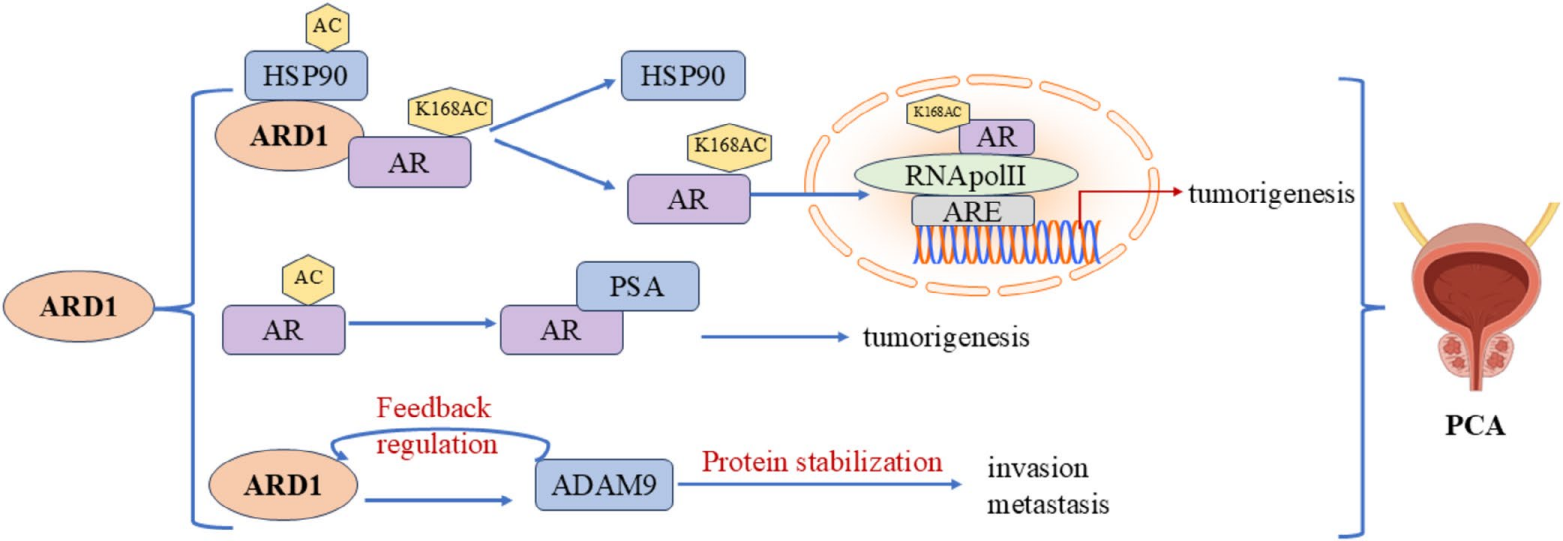
Molecular mechanisms of ARD1 in prostate cancer.

Prostate cancer (PCA) is the second most common cancer among men. In recent years, the acetylation of the androgen receptor (AR) has emerged as a crucial step in its activation, with ARD1 playing a key role in PCA progression (Figure 9). Katherine et al. [54] reported that upon androgen stimulation, ARD1 initially forms a trimeric complex with Hsp90 and AR. ARD1 is acetylated at the K618 site of AR, resulting in complex dissociation. Hsp90 stays in the cytoplasm while the acetylated AR translocates to the nucleus, binding to the Androgen Response Element (ARE) and RNA polymerase II, which initiates the transcription of prostate cancer‐related genes. This process not only activates the AR but also contributes to prostate tumorigenesis. Research revealed that ARD1 expression was significantly upregulated in prostate cancer cells and was regulated by androgens. Treatment with synthetic androgens (e.g., R1881) increased ARD1 expression, which AR‐specific siRNA or androgen inhibitors could inhibit. Depleting ARD1 using shRNA demonstrated that ARD1‐dependent expression is closely linked to prostate cancer cell proliferation, anchorage‐independent growth, and xenograft tumor formation, reinforcing the significance of ARD1 in PCA biology. Notably, ARD1 functions as an acetyltransferase for AR and a vital transcriptional regulator, playing a central role in regulating several AR target genes and creating a positive feedback loop. This mechanism indicates that ARD1 enhances gene transcription by facilitating AR binding to target promoters, thereby promoting prostate cancer progression [31]. Additionally, the interaction between ARD1 and ADAM9 is significant in androgen‐independent prostate cancer, as ARD1 can form a complex with ADAM9 to maintain its protein stability, subsequently enhancing the cells' invasive capacity. This mechanism operates independently of ARD1's acetyltransferase activity, with the AR‐ARD1 and ARD1‐ADAM9 interactions offering a new biological perspective on prostate cancer progression. In summary, ARD1's role in prostate cancer is intertwined through multiple mechanisms, including the acetylation and nuclear translocation of AR and its interaction with ADAM9 [55]. Disrupting these interactions could offer novel intervention strategies for treating both androgen‐dependent and ‐independent prostate cancer.

### 4.6 Molecular Mechanisms of ARD1 in Esophageal Cancer

**FIGURE 10:**
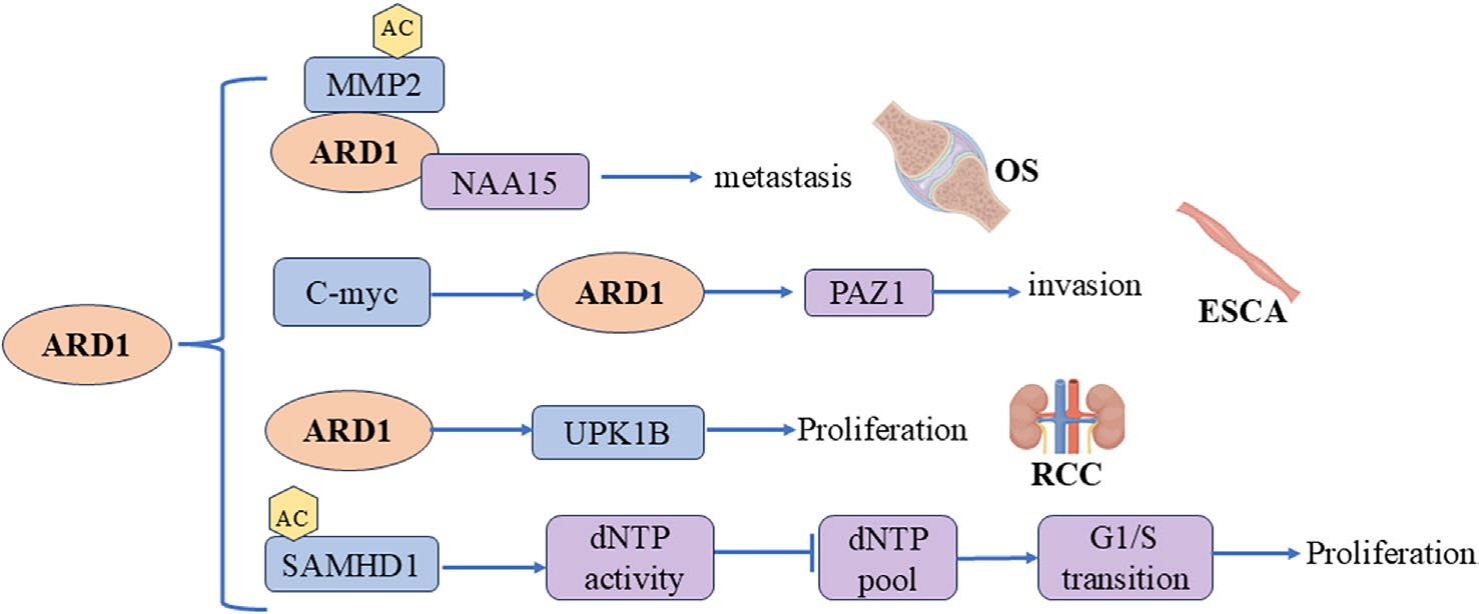
Molecular mechanisms of ARD1 in esophageal cancer, renal cell carcinoma and osteosarcoma.

The role of ARD1 in esophageal cancer is complex, exhibiting both tumor‐promoting and inhibitory effects (Figure 10). Its specific role may vary based on tumor type and cellular microenvironment, suggesting that future studies should explore the regulatory mechanisms of ARD1 across different cancers to clarify its potential as a therapeutic target.

Esophageal cancer (ESCA) is a malignant and aggressive disease that presents significant therapeutic challenges [56]. Pan et al. reported that ARD1 expression was elevated in esophageal cancer tissues compared to normal tissues, and high ARD1 expression correlated with poorer survival outcomes for patients with ESCA. Their study revealed that activation of the c‐Myc –ARD1 axis enhanced the invasive capacity of esophageal cancer cells. This indicates a potential positive regulatory role for ARD1 in enhancing the invasiveness of esophageal cancer cells [57]. Conversely, Wang et al. reported conflicting findings regarding ARD1's role in regulating tumorigenicity and invasiveness in ESCA. Their results indicated that ARD1 inhibits esophageal cancer cell proliferation, migration, and invasion, suggesting a tumor‐suppressive role for ARD1 in ESCA [58].

The difference between ARD1 expression in ESCA and its role in tumors may be because the two studies were conducted in different patient populations in different regions, and these populations may differ in genetic background, life, and other aspects, all of which may affect ARD1 expression and function. Pan et al. [57] used KYSE50, KYSE70, KYSE170, and KYSE510 cell lines to explore the role of the c‐Myc‐ARD1 axis in the invasion ability of esophageal cancer cells, while Wang et al. [58] used the TE‐1 cell line, and the role of ARD1 may be affected by the tumor microenvironment, such as tumor internal hypoxia, nutritional status, local inflammatory response, and other factors that may affect the function of ARD1. ARD1 may have a variety of biological functions; it may interact with different proteins in different signaling pathways, leading to different cell fate decisions; ARD1 may enhance the invasion ability of tumor cells by activating c‐myc under certain conditions and may exert tumor suppression by inhibiting certain signaling pathways under other conditions. These differences can lead to different results.

### 4.7 Molecular Mechanisms of ARD1 in Other Tumors

ARD1 plays a crucial role in various tumors, including renal cell carcinoma, osteosarcoma, hepatocellular carcinoma, and neurogliomas, by promoting proliferation, invasion, and metastasis. Its mechanisms involve key pathways (Figure 10).

Renal cell carcinoma (RCC) is a prevalent kidney malignancy ranking 3rd^rd^ among genitourinary cancers. Higher ARD1 expression was observed in renal cancer tissues compared to paracancerous tissues. Patients with elevated ARD1 levels displayed more advanced tumor stages. Moreover, ARD1 knockdown impeded the proliferative capacity of renal cancer cells, indicating a potential pro‐cancer role for ARD1 in renal cancer [59].

Osteosarcoma is the primary bone malignancy prevalent among children and adolescents. Chien et al. identified high expression of ARD1 in osteosarcoma tissues, which correlates with a poorer prognosis in patients. The complex of ARD1 and Naa15p acetylates and stabilizes the MMP‐2 protein, thus promoting the invasion and metastasis of osteosarcoma cells [60].

Liver cancer ranks sixth among malignant tumors and is the third leading cause of global cancer deaths [61]. Similar to many other malignancies, hepatocellular carcinoma exhibits overexpression of ARD1. Shim et al. investigated ARD1 expression in cancer tissues of 94 hepatocellular carcinoma patients, revealing a higher incidence of microvascular invasion in the high‐expression group. However, intra‐tumor *ARD1* mRNA levels did not correlate with the 5‐year recurrence rate or overall survival [62]. Lee et al. confirmed these findings, illustrating a proportional increase in ARD1 levels from low‐grade atypical hyperplasia to hepatocellular carcinoma and establishing a correlation between ARD1 expression and hepatocellular carcinoma progression [9]. However, the mechanism by which ARD1 regulates the development of hepatocellular carcinoma remains unclear.

Neurogliomas represent the most prevalent and aggressive tumors in the adult brain. Studies have indicated that gliomas exhibiting high ARD1 expression enhance the activity of critical biological processes linked to tumor progression, including cell proliferation and EMT. High ARD1 expression independently correlates with a poor prognosis of glioma [63].

It was shown that ARD1 catalyzes the acetylation of SAMHD1 at lysine 405 (K405) residues, thereby enhancing its ATPase activity. Acetylated SAMHD1 exhibited higher enzymatic activity, whereas the unacetylated K405R mutant lost this function. This mechanism suggests that the acetylation of SAMHD1 plays a key role in the G1/S phase transition of tumor cells and promotes cancer cell proliferation [24].

## 5 Conclusions and Perspectives

Despite extensive research, many aspects of ARD1 in tumors need further exploration. Up to now, therapeutic strategies against ARD1 have mainly focused on specific small molecule inhibitors or activators to regulate its acetylation activity. These compounds aim to regulate tumor cells' growth, survival, and metastasis by affecting the acetylation state of ARD1 and its substrate proteins. However, this field remains preliminary; no small molecule compounds directly targeting ARD1 have been reported. Its different substrates in various cancers mainly determine the dual effects of ARD1 in promoting and suppressing cancer. Therefore, further studies on the substrate profiles of ARD1, especially in different types of tumors, will contribute to a more comprehensive understanding of its biological functions, including the recognition of new substrate proteins and acetylation sites and how these acetylation events affect the behavior of tumor cells.

A key area requiring further investigation is the interaction between ARD1 and DNMT1 in cancer progression. DNMT1 plays a crucial role in maintaining DNA methylation status, and ARD1 enhances its binding to specific DNA regions, such as the E‐cadherin promoter, leading to the silencing of tumor suppressor genes. E‐cadherin's reduced expression is closely associated with increased invasiveness and metastasis in tumors. This interaction between ARD1 and DNMT1 underscores the importance of epigenetic regulation in tumor development and progression. Given that ARD1 may regulate other tumor suppressor genes and oncogenes through similar mechanisms, exploring its multi‐targeted epigenetic regulatory role could reveal new insights into cancer biology and open new therapeutic avenues. Specific inhibitors targeting ARD1 or DNMT1 could restore regular E‐cadherin expression, inhibit tumor invasiveness, and reduce metastasis, thus providing a promising strategy for treating cancers linked to E‐cadherin silencing.

Moreover, the metabolic state of cells in an inflammatory environment is altered, which affects ARD1 activity. This metabolic reprogramming may regulate ARD1's function and influence other immune and inflammatory responses, further complicating its role in tumorigenesis. Polymorphisms in the ARD1 gene may also influence cancer risk through changes in acetyltransferase activity. Variations in ARD1 activity could modify the epigenetic regulation of cancer‐related genes and contribute to tumor development. Exploring the impact of ARD1 gene polymorphisms on cancer could provide valuable insights into the molecular mechanisms underlying tumorigenesis and help identify novel targets for therapeutic intervention.

As for therapeutic strategies, developing small molecule inhibitors or activators targeting ARD1 remains a promising direction, but much work is needed. High‐throughput screening and drug design, combined with biochemical and cell biological approaches, will be key in identifying novel ARD1 substrates and validating their acetylation effects on tumor development. Gene editing techniques like CRISPR/Cas9 could manipulate ARD1 expression and study its effects on tumor‐related signaling pathways. This will help uncover the molecular mechanisms by which ARD1 regulates cancer progression. In vivo studies and clinical data analysis are necessary to evaluate the therapeutic potential of ARD1‐targeted interventions in various cancer types.

Finally, ARD1 expression has been reported to be elevated in multiple cancer types, suggesting that it may serve as a prognostic biomarker for cancer. By assessing the correlation between ARD1 expression levels and clinical outcomes, improving treatment options and clinical outcomes for cancer patients may be possible. Furthermore, ARD1 could emerge as a novel target for advanced cancer therapies. With continued research, we anticipate that the complex role of ARD1 in tumor development will become more apparent, ultimately paving the way for new anti‐tumor strategies and improving patient outcomes.

## Author Contributions

**Chunjiao Yu:** writing – original draft. **Hongtao Lei:** writing – review and editing. **Xuefei Hou:** investigation; visualization. **Shan Yan:** supervision; writing – review and editing.

## Ethics Statement

Approval of the research protocol by an Institutional Review Board: N/A.

Informed Consent: N/A.

Registry and the Registration No. of the study/trial: N/A.

Animal Studies: N/A.

## Conflicts of Interest

The authors of this manuscript have no conflicts of interest. None of the authors of this manuscript is a current editor or editorial board member of Cancer Science.

## Data Availability

Data sharing is not applicable to this article as no new data were created or analyzed in this study.
